# Selective recovery of europium from real acid mine drainage using modified Cr-MIL and SBA15 adsorbents

**DOI:** 10.1007/s11356-024-34566-2

**Published:** 2024-08-08

**Authors:** Charith Fonseka, Seongchul Ryu, Youngwoo Choo, Jaya Kandasamy, Lena Foseid, Harsha Ratnaweera, Saravanamuthu Vigneswaran

**Affiliations:** 1 Department of Civil and Environmental Engineering, Faculty of Engineering and IT, University of Technology Sydney, P.O. Box 123, Broadway, Sydney, Ultimo, NSW 2007 Australia; 2 Department of Building and Environmental Technology, Faculty of Sciences & Technology (RealTek), Norwegian University of Life Sciences, P.O. Box N-1432, Oslo, Norway

**Keywords:** Adsorbent, Acid mine drainage, Resource recovery, Rare earth elements

## Abstract

The successful adoption and widespread implementation of innovative acid mine drainage treatment and resource recovery methods hinge on their capacity to demonstrate enhanced performance, economic viability, and environmental sustainability compared to conventional approaches. Here, an evaluation of the efficacy of chromium-based metal–organic frameworks and amine-grafted SBA15 materials in adsorbing europium (Eu) from actual mining wastewater was conducted. The adsorbents underwent comprehensive characterization and examination for their affinity for Eu. Cr-MIL-PMIDA and SBA15-NH-PMIDA had a highest Langmuir adsorption capacity of 69 mg/g and 86 mg/g, respectively, for an optimum level of pH 4.8. Preferential adsorption tests followed using real AMD collected at a disused mine in the north of Norway. A comparative study utilizing pH-adjusted real AMD revealed that Cr-MIL-PMIDA (88%) exhibited slightly higher selectivity towards Eu compared to SBA15-NH-PMIDA (81%) in real mining wastewater. While Cr-MIL-PMIDA displays excellent properties for the selective recovery of REEs, practical challenges related to production costs and potential susceptibility to chromium leaching make it less appealing for widespread applications. A cost–benefit analysis was then undertaken to quantify the advantages of employing SBA15-NH-PMIDA material. The study disclosed that 193.2 g of EuCl_3_ with 99% purity can be recovered by treating 1000 m^3^ of AMD.

**Supplementary Information:**

The online version contains supplementary material available at 10.1007/s11356-024-34566-2.

## Introduction

Rare earth elements (REEs) have gained critical importance globally, driven by the escalating demand for high-tech products, renewable energy technologies, and military applications. Nations worldwide are actively seeking stable and continuous access to REEs. There continues to be significant interest and funding in research and development focused on mining, processing, and recycling to promote sustainable supply sources. Europium (Eu) stands out among the REEs for its high cost and scarcity, comprising 0.1% w/w in monazite and bastnaesite ores (Kumari et al. 2019). Consequently, financial constraints have led to europium being predominantly mined as a byproduct. Eu finds widespread use in phosphor production, alloys, additives, and serves as a critical raw material for the development of smart devices, ranging from high-resolution color screens to circuitry (Iloeje et al. 2022; Wang et al. 2020). In fast breeder nuclear plants, europium oxide is extensively used in control rods to absorb neutrons. Therefore, the search for alternative sources of europium can drive advancement of technology and industry (Zhang et al., 2022). This is essential to ensure a sustainable and environmentally friendly supply chain.

Various technologies, including chemical precipitation (Ambaye et al. 2020; Fonseka et al. 2023; Tao et al. 2023), solvent extraction (Arrachart et al. 2021; Seyyed Alizadeh Ganji et al. 2016), ion exchange (Felipe et al. 2021; Khawassek et al. 2019), adsorption (Callura et al. 2018; Cerrahoğlu et al. 2017; Kayan. A., 2019; Ryu et al. 2021a; Smith et al. 2016), and membrane filtration (Murthy and Choudhary 2011; Naidu et al. 2019), are extensively researched for REE recovery. While these technologies offer distinct advantages, they also present unique challenges such as the high cost of solvents, matrix interferences, blocking of membrane pores, and the need for waste management and disposal of the residue. Therefore, adsorption is considered the best potential method for high efficiency and low cost (Callura et al. 2018). Adsorption is a process where REE ions adhere to the surface of solid adsorbents. This method is notable for its versatility and effectiveness in treating low-concentration REE solutions. Fonseka et al. (2021) studied the use of functionalized chromium-based metal–organic framework for selective recovery of europium. However, high cost of production and susceptibility to chromium leaching were found to be inherent concerns. Cerrahoğlu et al. (2017) and Ryu et al. (2021a) investigated the development of functionalized mesoporous silica-based adsorbents, which offer high surface area and tailored functionalities for enhanced REE adsorption.

However, most traditional adsorbents exhibit limited selectivity towards REE recovery (Naidu et al. 2019). Hence, modification with favorable functional groups is necessary to enhance performance. Metal–organic framework (MOF) materials functionalized with specific ligands have demonstrated high affinity towards REEs (Abdel-Magied et al. 2019; Fonseka et al. 2021). Notably, functionalized chromium-based MOF (Cr-MIL-PMIDA) selectively recovered Eu from the leachate of zinc ore due to the formation of strong chelating complexes with phosphonic and carboxylate ligands (Lee et al. 2018; Fonseka et al. 2021). However, the high cost of production, coupled with concerns over long-term stability, hinders the application of these MOFs at an industrial scale. Mesoporous silica materials (SBA15-NH-PMIDA) offer excellent tunability and modification capabilities, making them an attractive, low-cost alternative (Fonseka et al. 2023; Rivas-Sanchez et al. 2022). There has been no previous study done to analyze the performance of these two materials in selectively adsorbing Eu using real acid mine drainage. This is the first time; these two adsorbents were tried with real acid mine drainage which has several foreign elements along with Eu. Hence, it is important to analyze both types of MOFs to evaluate the scalability for industrial applications with real acid mine drainage to recover Eu based on adsorption performance, chemical stability, and cost.

In this investigation, the main objective was to assess how well functionalized MOFs, SBA15-NH-PMIDA, and Cr-MIL-PMIDA can selectively recover Eu from real mining wastewater. The N-(phosphonomethyl) iminodiacetic acid (PMIDA) ligand was selected to modify both materials due to its reported high affinity towards REEs in previous studies (Lee et al. 2018; Fonseka et al. 2021). First, detailed adsorption studies for Eu removal were carried out using synthetic solutions at varying levels of pH to determine optimum conditions. AMD samples obtained from a disused mining site in northern Norway were then employed in selective adsorption experiments. Finally, detailed physical and chemical analyses of both materials were carried out to establish adsorption mechanisms.

## Material and methods

### Materials

Chromium(III) nitrate nonahydrate (Cr(NO_3_)_3_·9H_2_O, 99%), 2-aminoterephthalic acid (H_2_BDC-NH_2_, 99%), N-(phosphonomethyl) iminodiacetic acid (PMIDA, 95%), N,N-dicyclohexylcarbodiimide (DCC, 99%), methyl alcohol (CH_3_OH, 99.8%), N,N-dimethylformamide (DMF, 99%), and toluene (anhydrous, 99.8%) were used for synthesis of Cr-MIL-PMIDA. Furthermore, tetraethyl orthosilicate Si(OC_2_H_5_)_4_ (TEOS, Sigma-Aldrich, 98%), poly(ethylene glycol)-block-poly(propylene glycol)-block-poly(ethylene glycol) (P123), (3- aminopropyl) triethoxysilane (APTES, Sigma-Aldrich) were used to synthesize SBA15. Synthetic Eu solutions were prepared using europium (III) nitrate pentahydrate (Eu(NO_3_)_3_·5H_2_O, 99.9%). pH adjustment was achieved using 0.1 M HCl solution and potassium hydroxide (KOH, 98%, Sigma-Aldrich). Ultra-pure Milli Q water was obtained by filtering through Millipak Express 40 Filter (0.22 µm membrane filter, 18 M Ω cm). Sigma-Aldrich supplied all chemicals in this study and were used without further purification.

### Cr-MIL-PMIDA

**Fig. 1:**
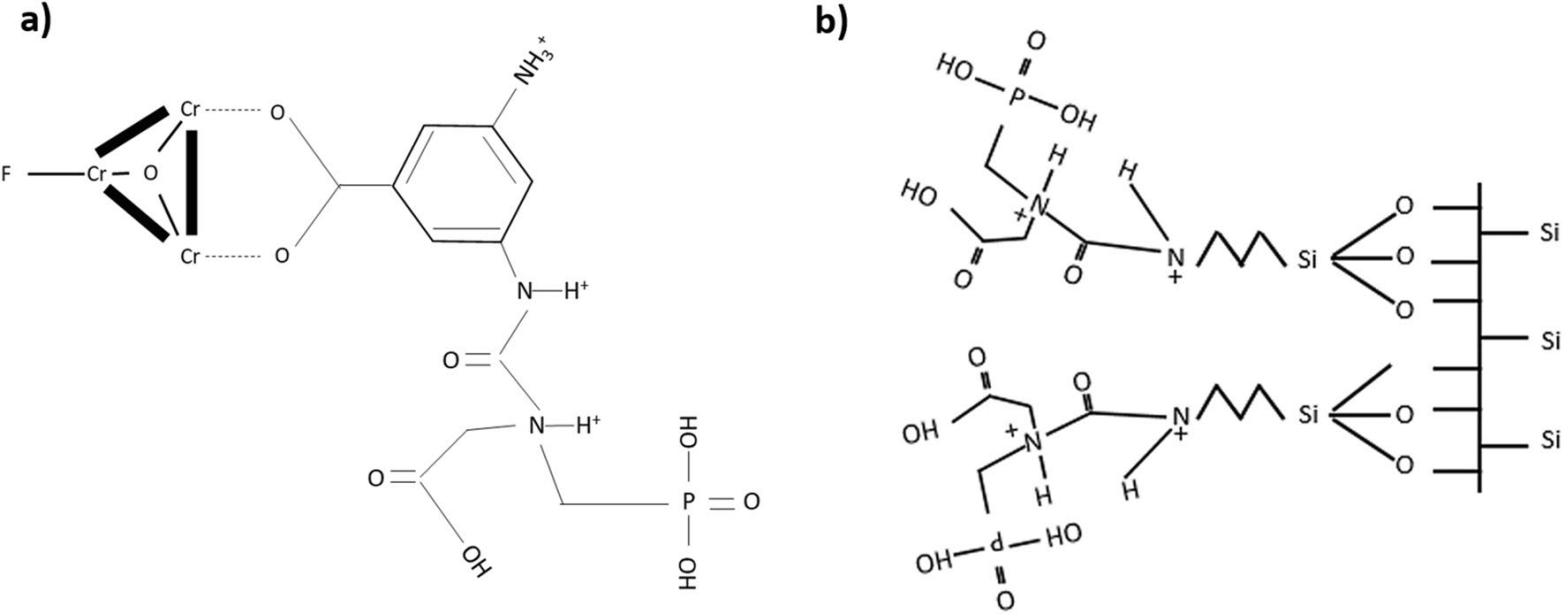
Open structures of prepared (**a**) Cr-MIL-PMIDA and (**b**) SBA15-NH-PMIDA (Fonseka et al. 2021, 2023)

The preparation of Cr-MIL-PMIDA MOF was done according to the method reported by Fonseka et al. (2021). First, 0.58 g of 2-aminoterephthalic acid, 1.25 g of chromium(III) nitrate nonahydrate, and 17.5 ml of Milli Q water were mixed at room temperature (25 ± 1 ℃). The mixture was stirred for 3 h at 25 ± 1 ℃ and then transferred to an autoclave. The autoclave was heated to 150 ℃ for 24 h and allowed to cool to room temperature. The dark green Cr-MIL-NH2 precipitate was then filtered and washed with Milli Q water and ethyl alcohol. The washed precipitate was then dried at 80 ℃ for 12 h to obtain Cr-MIL-NH2 powder. Further modification was performed by mixing Cr-MIL-NH2, PMIDA, and DCC at a 1:1.5:2 molar ratio in 80 ml of DMF. The solution was refluxed at 150 ℃ for 48 h and then allowed to cool down to room temperature. The solution was then filtered to obtain light green Cr-MIL-PMIDA precipitate, which was washed with toluene and ethanol, and dried at 100 ℃ for 12 h (Fig. 1).

### SBA15-NH-PMIDA

Modified SBA15 was prepared via a hydrothermal reaction reported by Fonseka et al. (2023). One hundred twenty milliliters of 2 M HCl, 23 ml of deionized water, and 3 g of P123 were first mixed at 25 ± 1 ℃. Then, 6.5 g of TEOS was added, and the mixture was transferred to an autoclave and heated to 35 ℃ for 20 h, followed by 24 h at 100 ℃. The precipitate was then filtered, dried at 80 ℃ for 12 h and calcinated at 550 ℃ for 3 h in atmospheric air to obtain SBA15 powder. Then, 1.0 g of SBA15 and 1 ml of APTES were dispersed in 100 ml of dry toluene. The mixture was refluxed at 110 ℃ for 10 h and the resulting SBA15-NH_2_ precipitate was filtered washed with ethanol, and dried at 70 ℃ for 12 h. Powdered SBA15-NH_2_, PMIDA, and DCC were then mixed at a 1.5:1.5:2 molar ratio in 80 ml of DMF. The solution was refluxed at 150 ℃ for 48 h, cooled to room temperature, and filtered to obtain whitish SBA15-NH-PMIDA precipitate. The product was washed with toluene and ethyl alcohol, then dried in a convection oven at 100 ℃ for 12 h to obtain powdered SBA15-NH-PMIDA.

### Adsorbents

#### Structure (XRD)

The crystalline structure of modified chromium-based metal–organic framework and modified SBA15 materials analyzed using Bruker D8 Discover diffractometer. The experiment was conducted at 40 kV using Cu Kα1 radiation. The analysis spanned the temperature range of 0 to 55 °C, with a controlled scan rate of 2°/min.

#### Surface area and pore size distribution

The physical characteristics of the two adsorbents were investigated through nitrogen adsorption and desorption studies. These examinations were carried out using a Nanoporosity Mirae instrument, with measurements performed at a temperature of 77 K. Surface area of the specimens were measured using Brunauer–Emmett–Teller (BET) with the range 0 < P/Po < 1.0. Pore distribution and volume was measured with the Barrett–Joyner–Halenda (BJH) method.

#### Surface morphology element analysis

The surface morphology of the adsorbents in terms of chemical bonds and surface vibrations was measured by a Nicolet 6700 FTIR spectrometer. Zeiss Supra 55VP instrument was used to obtain scanning electron microscopy (SEM) images. Energy-dispersive X-ray spectrometer (EDS) mapped the adsorbent’s elemental composition.

### Adsorption studies

#### Equilibrium

Single-solute europium (Eu) adsorption experiments were first conducted comparing adsorption capacities of Cr-MIL-PMIDA and SBA15-NH-PMIDA. The experiments were carried out at optimal pH levels (pH = 4.75 ± 0.25) determined in prior studies (Fonseka et al. 2021, 2023). Initial concentrations of Eu ranged from 2.5 to 30 mg/l to calculate the maximum adsorption capacity of the two materials. All samples were agitated on a flat shaker (100 rpm) operating for 24 h at room temperature (24 ± 1 °C). Inductively coupled plasma mass spectrometry (ICP-MS, Agilent 7900, USA) was used for measurements of initial and equilibrium concentrations of Eu. The equations used for the modelling of equilibrium isotherms are given in Eqs. 1, 2 and 3.

1 $$\text{Adsorption Capacity}: {Q}_{e}=\frac{\left({C}_{i}-{C}_{e}\right) V}{m}$$

2 $$\text{Langmuir Isotherm}:{ Q}_{e}= \frac{{Q}_{m}b{C}_{e}}{1+b{C}_{e}}$$

(Langmuir 1918)

3 $$\text{Freundlich Isotherm}: {Q}_{e}= K{{C}_{e}}^\frac{1}{n}$$

(Haring 1926)

where,

*C*_i_: Eu initial concentration (mg/l).

*C*_e_: Eu equilibrium concentration (mg/l).

*m*: adsorbent mass (g).

*Q*_m_: Maximum adsorption capacity (mg/g).

1/n: Dimensionless constant.

*V*: solution volume (l).

*Q*_e_: equilibrium adsorption capacity (mg/g).

*b*: Langmuir constant (L/mg).

*K*: Freundlich Constant (g^1−n^ L^n^g^−1^).

### Selective adsorption studies using real mining wastewater

The aim of this investigation was to assess and compare the selective adsorption capacities of Cr-MIL-PMIDA and SBA15-NH-PMIDA utilizing real mining wastewater. AMD samples from a disused mine site in north of Norway were collected for this study. AMD samples were first measured for pH and concentrations of dissolved metal. Subsequently, the pH was adjusted using 0.01 M NaOH and used for determining the optimal conditions. This set the conditions for the subsequent selective adsorption investigation. Multimeter (HQ40d, HACH, USA) was used to measure pH. Selective adsorption experiments were performed employing predefined amounts of powdered SBA-NH-PMIDA. In experiments, two 50 ml beakers holding pH-adjusted real AMD and 0.04 g of the adsorbents were used. Samples were agitated on flat shaker (100 rpm) operating for 24 h at room temperature (24 ± 1 °C). This allowed sufficient time for the adsorption process to reach equilibrium, ensuring accurate and consistent results. Inductively coupled plasma-mass spectrometry (ICP-MS, Agilent 7900, USA) measured the dissolved cation concentrations before and after adsorption.

### Reusability study

The reusability of SBA15-PMIDA and Cr-MIL-PMIDA was assessed over ten sequential cycles of adsorption and desorption using real mining wastewater. For regeneration, 0.01 M HCl was used as the stripping agent. All used adsorbents were first filtered and mixed with 0.01 M HCl. Then, samples were agitated on a flat shaker (100 rpm) for 2 h at room temperature (24 ± 1 °C) to desorb Eu ions. Finally, adsorbents were washed with Milli Q water and dried in the oven at 100 °C for 12 h before the next use.

## Results and discussion

### Adsorbent characterization

#### Crystal structure

**Fig. 2:**
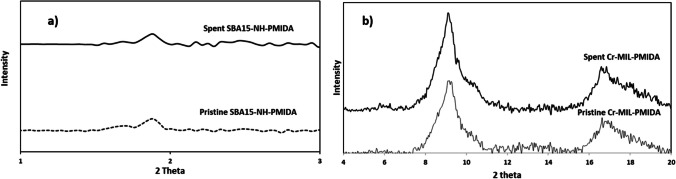
XRD patterns of (**a**) SBA15-NH-PMIDA and (**b**) Cr-MIL-PMIDA

The X-ray diffraction (XRD) patterns of Cr-MIL-PMIDA and SBA15-PMIDA closely resemble those documented in previous studies, providing confirmation of the successful synthesis (Lee et al. 2018; Fonseka et al. 2021, 2023). The identical XRD patterns observed for both materials, consistent with earlier studies, underscore the effective preservation of the crystal structure of the adsorbent during the modification with PMIDA groups. As illustrated in Fig. 2, Cr-MIL-PMIDA maintains its peak in the 2θ = 9° (± 1°) region that attest the preservation of the crystal structure throughout the synthesis process. The XRD pattern of SBA15-NH-PMIDA reveals distinct peaks that characteristically align with mesoporous silica’s hexagonal structure (Ryu et al. 2019). The (1 0 0) peak is a reflection that corresponds to the periodic arrangement of the mesopores. This peak arises from the regular arrangement of pores in the mesoporous silica. The intensity and sharpness of this peak reflect the degree of order in the mesoporous structure (Ryu et al. 2019). The reduction in the peak at (1 0 0) for SBA15-NH-PMIDA signifies a decrease in the mesoscopic order of the structure (Da’na and Sayari, 2011), potentially induced by grafted phosphonic groups leading to pore reduction.

#### SEM and EDS analysis

Scanning electron microscopy (SEM) was employed to analyze the structural characteristics, surface morphology, and elemental composition of pristine SBA15-NH-PMIDA and Cr-MIL-PMIDA. SEM images of SBA15-PMIDA reveal a well-ordered, 2D hexagonal structure with uniform arrangement and consistent spacing, characteristic of mesoporous materials (Ryu et al. 2021b). These indicate that the modified adsorbents retained a properly ordered structure similar to virgin SBA15, consistent with findings reported in previous studies (Ryu et al. 2019). Similar observations were made for Cr-MIL-PMIDA, where particles were observed to be agglomerated (Fonseka et al. 2021). However, discrete octahedral formations are visible even after modification with PMIDA, highlighting the structural and thermal stability of the material (Fig. S1).

#### Chemical properties

FTIR spectroscopy was conducted to investigate the chemical functionalities and characteristics of the prepared adsorbents (Fig. S2). The Cr-MIL-PMIDA showed a strong stretching band for amine (N–H) identified at 3350 cm^−1^ (Hwang et al. 2008). This is attributed to either N–H bond present in the amide bonding, or unreacted amine ligands on the surface of Cr-MIL-PMIDA. The small peak appearing at 2940 cm^−1^ is attributed to the asymmetric stretching of the aliphatic C–H bond present in the organic linker (Bai et al. 2015). An additional peak around 1610 cm^−1^ is attributed to the carbonyl (C = O) stretch. The split peaks at 1400 cm^−1^ are due to C–O stretching vibrations, with one peak influenced by hydrogen bonding and the other representing a free C–O bond (Bai et al. 2015). The spectrum exhibited minor peaks in the ranges 1100–900 cm^−1^ that can be assigned to –PO_3_H_2_ stretching modes, corroborating the functionalization of the Cr-MIL MOF with PMIDA moiety (Lee et al. 2018). SBA15-NH-PMIDA sample exhibited FTIR spectrum similar to that of Cr-MIL-PMIDA with exception of strong peaks at 1050 and 930 cm^−1^ that can be attributed to the symmetrical and anti-symmetric vibration bands of siloxane (Si–O–Si) bond (Kayan et al., 2022), consistent with typical FTIR spectra of SBA15 (Ryu et al. 2019; Dolatyari et al. 2016). The FTIR spectra of SBA15 confirm that the surface functionalization with PMIDA was successfully performed.

#### Surface area and pore size distribution

The nitrogen adsorption and desorption isotherms of Cr-MIL-PMIDA and SBA15-NH-PMIDA samples are presented in Fig. S3. The graph for Cr-MIL-PMIDA exhibits a trend very similar to the previously reported results (Lee et al. 2018). The BET surface area of the modified MOF was determined to be 1049 m^2^/g, with a mean pore diameter of 2.15 nm. This represents a surface area over 9 times higher and a pore size 3 times smaller compared to the modified SBA15. The modification of SBA15 led to a decrease in the amount of nitrogen adsorbed, possibly attributed to pore-blocking with grafted ligands. The clear hysteresis displayed in the adsorption/desorption curves indicates the maintenance of the mesoporous structures of SBA15 despite chemical modification with PMIDA. Nevertheless, the BET surface area decreased from 826 to 113 m^2^/g, and the total pore volume decreased from 1.26 to 0.36 cm^3^/g (Hernández-Morales et al. 2012; McManamon et al. 2012). Due to the chemical ligands grafted into the pores of SBA15, the size of mesopores, as per the BJH theory, decreased from 8.16 to 7.16 nm (Table S1) (Mureseanu et al. 2008; Prasetyanto and Park 2008). As depicted in the BJH pore size distribution (PSD) graphs (Fig. S5), the peak position of the modified SBA-15 shifted slightly to the left while still maintaining the peaks (Majda et al. 2016). This suggests the successful implementation of the SBA-15 modification.

### Adsorption study

#### Equilibrium

Equilibrium studies were undertaken using synthetic Eu solutions, providing researchers with precise control over the solution’s composition and ensuring a well-defined and consistent environment. This control is essential for a reliable comparison of the adsorption characteristics of the two adsorbents, eliminating the complexities introduced by variables present in real waste. Langmuir and Freundlich isotherm models were employed to analyze and compare the adsorption capacities of SBA15-NH-PMIDA and Cr-MIL-PMIDA. These models offer valuable insights into the adsorption behavior of the materials, aiding in the understanding of how adsorbates interact with the surfaces of the adsorbents under specific conditions.

**Fig. 3:**
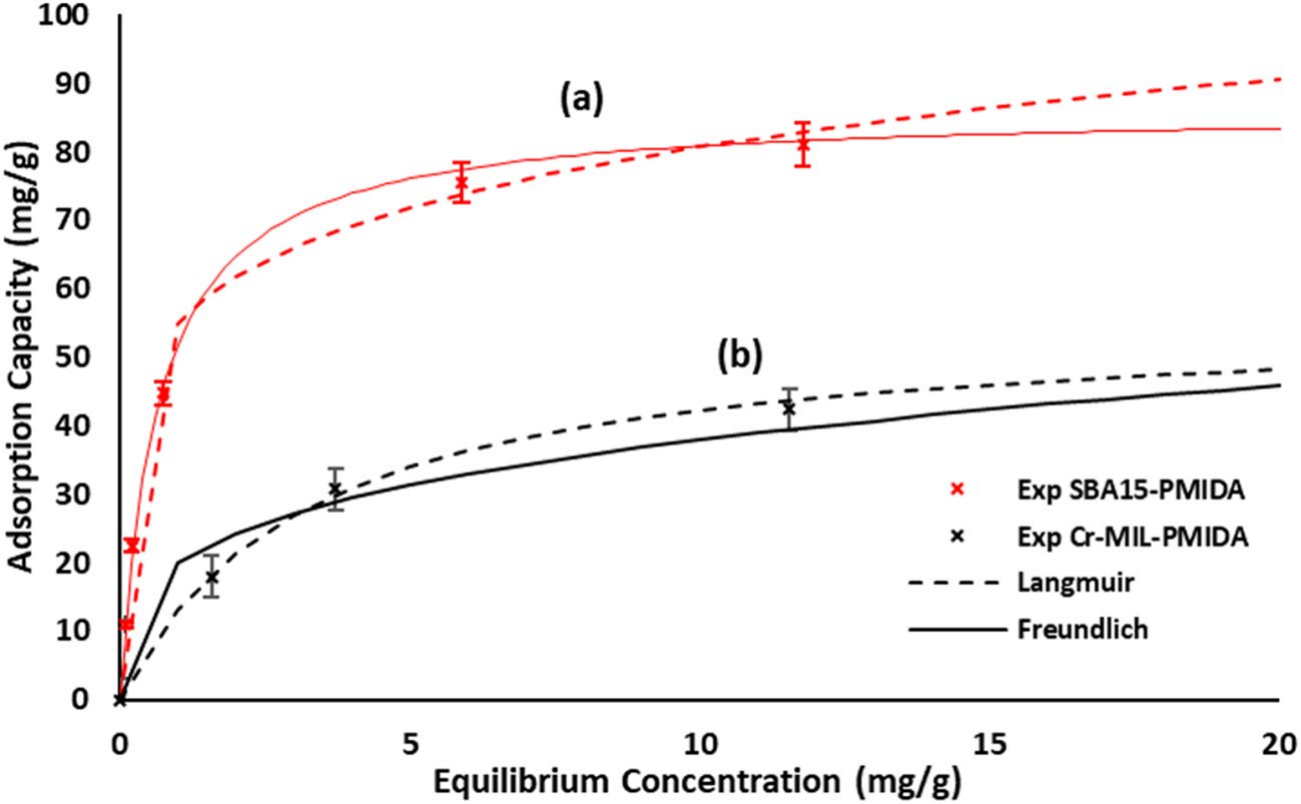
Isotherm modelling for adsorption of Eu on Cr-MIL-PMIDA and SBA15-PMIDA (0.01 g of adsorbent dose in 50 ml for 24-h equilibrium time, pH = 4.75 ± 0.1)

As depicted in Fig. 3, the adsorption capacity displayed a non-linear pattern for both adsorbents, indicating a dependence on the initial concentration of europium. This observation suggests that the adsorption process is not strictly linear and may be influenced by factors such as surface saturation or competition for adsorption sites at higher initial concentrations. Table 1 summarizes the isotherm parameters calculated from the models. The Langmuir model, with an *R*^2^ value exceeding 0.98, provided the best fit to the experimental data. This high goodness of fit implies the presence of homogeneous binding sites for the adsorption of europium on Cr-MIL-PMIDA and SBA15-NH-PMIDA. The highest Langmuir adsorption capacity for uptake of Eu on Cr-MIL-PMIDA and SBA15-PMIDA was calculated to be 69.14 mg/g and 86.11 mg/g, respectively.

**Table 1:** Adsorption Isotherm parameters

	Langmuir			Freundlich		
	*Q*_*m*_ (mg/g)	*b* (L/mg)	*R*^2^	*K*_*F*_ (g^1−n^ L^n^g^−1^)	*n*	*R*^2^
SBA15-PMIDA	86.11	1.52	0.98	54.89	5.99	0.97
Cr-MIL-PMIDA	69.14	1.77	0.99	35.46	4.11	0.94

#### Recovery of Eu from real acid mine drainage (AMD)

While synthetic solutions provide controlled and well-defined conditions, it is crucial to subsequently validate findings with real waste to ensure the relevance and applicability of the results in practical scenarios. This study primarily aimed to evaluate the potential for selectively recovering Eu from AMD using both Cr-MIL-PMIDA and SBA15-PMIDA. Initially, AMD collected from a disused mine located in the north of Norway was subjected to a thorough chemical composition analysis (refer to Table 2).

**Table 2:** Chemical composition of AMD obtained from abandoned mining site in Norway (pH = 2.1 ± 0.1)

Dissolved ions	Na	Mg	Al	S	Ca	Cr	Mn	Fe	Ni	Cu	Zn	Eu
Concentration (mg/l)	36	218	168	1275	187	0.38	6.29	618	0.51	62	39	3.1

AMD typically contains elevated sulfur content (as SO_4_^2−^), transition metals, and multivalent ions (Fe, Mg, Al, Ca, Cu, Zn) with low pH (2.0 ± 0.1). REE were found to be present in low concentrations, with europium (Eu) being the predominant rare earth element at 3.1 mg/l (refer to Table 2). The selective recovery of Eu using Cr-MIL-PMIDA and SBA15-PMIDA faces competition for adsorption sites due to the high concentrations of trivalent Fe and Al in AMD (Kavun et al. 2021). Therefore, careful consideration and optimization of operating conditions, such as pH adjustment, are essential to address these obstacles, increase Eu selectivity, and enhance recovery.

#### Influence of pH on solubility of dissolved metals in AMD

**Fig. 4:**
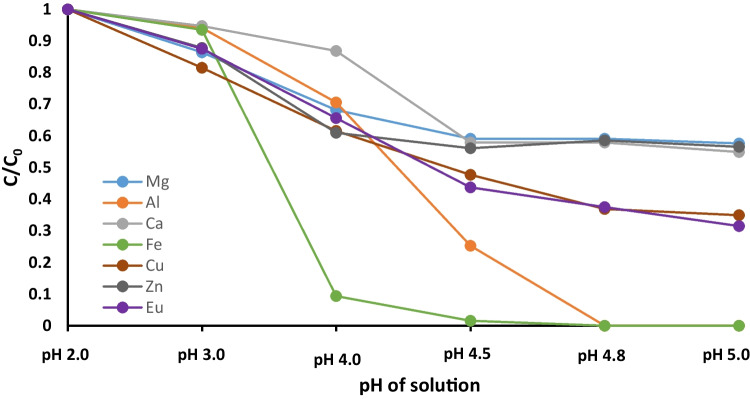
Precipitation of dissolved metals in AMD through pH correction (where C, concentration at initial pH, C_0_, concentration at adjusted pH)

The objective here was to investigate how pH affects the leaching and solubility of metals in acid mine drainage (AMD) (see Fig. 4). Sodium hydroxide (NaOH), a widely used industrial alkaline agent, was utilized to adjust AMD’s pH from 2.0 to 5.0 (Tao et al. 2023). Optimum conditions were isolated by measurements of dissolved metals at different pH values. Trivalent Fe and Al, present in high concentrations, can adversely affect the selectivity of Eu by SBA15-NH-PMIDA (Fonseka et al. 2023). The ionic properties of Fe^3+^ and Al^3+^ are similar to Eu^3+^, and these ions are able to occupy functional groups that lie on the surface of the adsorbent, thereby reducing SBA15-NH-PMIDA’s capacity to adsorb Eu. Trivalent Fe^3+^ and Al^3+^ ions are positively charged, akin to Eu^3+^. Electrostatic interactions between these positively charged Fe^3+^ and Al^3+^ ions and the negatively charged functional groups of SBA15-PMIDA may disrupt the selective adsorption of Eu ions (Kavun et al. 2021). This interference can lead to the decrease in the adsorbent’s selectivity for Eu. Precipitation of Fe and Al is favored at pH between 4.5 and 5.0 with over 99% removal, while approximately 38% of Eu remained dissolved (refer to Table S2). The cause is the low solubility limit of Fe^3+^ and Al^3+^ in this pH range (Miranda et al. 2022).

These observations confirm benefit of altering the pH of AMD to 4.75 ± 0.1, as it resulted in the precipitation of nearly all Fe/Al and aligned with the optimum pH range for the maximum uptake of Eu onto SBA15-NH-PMIDA. However, once pH was increased to 5.0, more europium was found to precipitate. This reduces the efficiency of Eu recovery as it decreases the availability of free Eu ions in the solution that can be adsorbed. Consequently, pH 4.75 ± 0.1 was chosen for all selective adsorption experiments to optimize Eu availability.

#### Comparison of selective adsorption of Eu between Cr-MIL-PMIDA and SBA15-NH-PMIDA

A comparative analysis of the selectivity for europium (Eu) uptake between Cr-MIL-PMIDA and SBA15-NH-PMIDA was conducted with real AMD obtained from northern Norway (refer to Table 2). The study aimed to discern and evaluate the differential capabilities of these two materials in terms of their preferential adsorption of Eu ions. Cr-MIL-PMIDA, characterized by its metal–organic framework nature, exhibited well-defined crystalline structures with inherent porosity and adjustable functionalities.

**Fig. 5:**
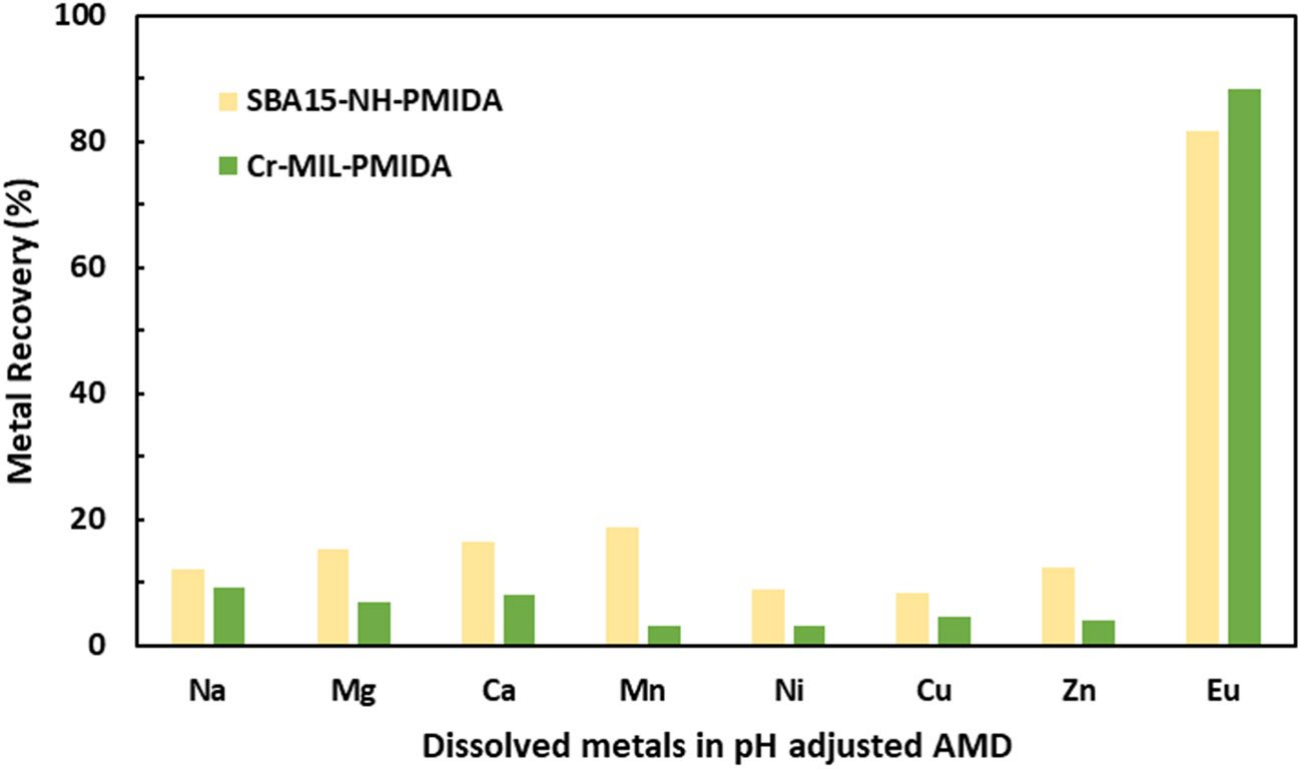
Recovery of Eu from pH-adjusted real AMD using Cr-MIL-PMIDA and SBA15-NH-PMIDA (experimental condition, 50 ml AMD volume, 0.8 g/l adsorbent dose, 24-h equilibrium time, pH = 4.75 ± 0.1)

As depicted in Fig. 5, Cr-MIL-PMIDA exhibits superior selectivity towards Eu (88%) compared to SBA15-NH-PMIDA (81%). Since the two materials possess the same PMIDA functional ligands, differences in selectivity can be attributed to variations in physical properties, such as pore size, surface area, and structural characteristics (Callura et al. 2018). These physical properties influence the interactions between the ligands and the adsorbed species, ultimately affecting the materials’ adsorption behavior.

Coordination sites within the Cr-MIL-PMIDA structure demonstrated significant affinity for Eu ions due to tailored ligand properties and metal links, resulting in strong adsorption. SBA15-NH-PMIDA, characterized by its mesoporous silica nature, possesses a well-ordered, large-pore structure with accessible surface area. According to Table S1, Cr-MIL-PMIDA has a larger surface area and pore volume (more than 2 folds). However, SBA15-NH-PMIDA has a larger pore size (9.16 nm) compared to Cr-MIL-PMIDA (2.15 nm). The higher adsorption capacity of Eu in single-solute form observed with SBA15-NH-PMIDA (86.21 mg/g) compared to Cr-MIL-PMIDA (69.14 mg/g) can be attributed to the combination of a larger pore size and the structural characteristics that promote multi-layer adsorption and the formation of strong complexes. On the other hand, Cr-MIL-PMIDA’s size-selective adsorption and potentially stronger ligand–metal interactions might contribute to its slightly lower adsorption capacity. The interplay of these factors underscores the importance of understanding the materials’ structural and surface properties when interpreting and optimizing their adsorption performance.

The larger pore size of SBA15-NH-PMIDA allows for increased spatial freedom within the pores, potentially enabling more diverse coordination and binding interactions between the adsorbent’s functional groups and various metal species (Lee et al. 2018). Cr-MIL-PMIDA has smaller pore sizes which restrict the entry of larger metal ions or complex species, leading to size-selective adsorption (Fonseka et al. 2021). The difference in pore sizes between SBA15-NH-PMIDA and Cr-MIL-PMIDA can significantly impact selectivity. Fonseka et al. (2023) have mentioned that the larger pore size of SBA15-NH-PMIDA accommodates larger metal species and complex molecules, broadening its selectivity profile. In contrast, Cr-MIL-PMIDA pore sizes which are smaller restrict the entry of larger species, contributing to its size-selective adsorption (Lee et al. 2018; Fonseka et al. 2021). Although both materials contain the same PMIDA ligand, the arrangement of the ligands on the surface of the material can differ. Even slight variations in the orientation or density of ligands can lead to differences in the steric effects and electrostatic interactions with adsorbed species (Lu et al. 2021).

#### Cyclic adsorption/desorption results

The ability to reuse SBA15-PMIDA and Cr-MIL-PMIDA was evaluated over ten sequential cycles of adsorption/desorption using real mining wastewater. For regeneration, 0.01 M HCl served as the stripping agent, followed by a thorough wash with Milli Q water. During this process, at low pH, the functional ligands protonated and the adsorbent surface obtained a positive charge (Fonseka et al. 2021, 2023). This repelled Eu^3+^ ions and induced their desorption into the HCl solution.

**Fig. 6:**
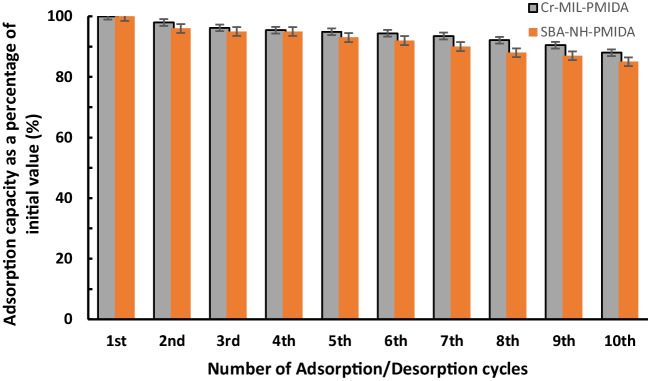
Cyclic adsorption tests for Eu^3+^ recovery

Figure 6 shows both SBA15-PMIDA and Cr-MIL-PMIDA maintained over 85% of their original adsorption capacity over ten cycles of adsorption and desorption with a standard error of 1.7% and 1.9%, respectively. This suggests that the regeneration process caused little loss of functional groups. These results highlight that modified SBA15 exhibits superior adsorption capacity and reusability, rendering it more suitable for industrial applications. Notably, previous studies have similarly illustrated how stable Cr-MIL-PMIDA was over repeated cycles of adsorption/desorption (Fonseka et al. 2021, 2023).

#### Cost–benefit analysis

When assessing the performance of Cr-MIL-PMIDA and SBA15-NH-PMIDA, SBA15-NH-PMIDA demonstrated superior adsorption capacity and reusability. Additionally, SBA15 materials can be synthesized at a lower cost compared to chromium-based metal–organic frameworks. This is due to high costs of raw materials required for MOF synthesis (Fonseka et al. 2021). Consequently, SBA15-NH-PMIDA was selected for a high-level costing of basic functions to assess the viability of recovering Eu from AMD. Conducting a cost–benefit analysis for a novel adsorbent such as SBA15-NH-PMIDA is crucial to evaluate economic feasibility and potential benefits of its application in the industry.

Adsorbing Eu from the concentrate obtained through a membrane process can provide increased selectivity (Fonseka et al. 2023). The specific characteristics of the membrane process, combined with the properties of the adsorbent material, can contribute to enhanced selectivity in capturing and separating Eu from the concentrated solution. This analysis was performed by calculating the costs and potential revenue of treating 1000 m^3^/day of AMD with chemical composition listed in Table 2.

As depicted in Fig. S5, NaOH is first used for pH correction. Subsequently, the resultant supernatant water is passed through the membrane process. Assuming 1.3 kg of SBA15-NH-PMIDA is available and based on its maximum adsorption capacity of 86 mg/g, Eu available in over 1000 m^3^ of AMD can be recovered. Based on an 80% water recovery rate, around 800 m^3^ of clean water can be recovered from this process. According to the calculation, approximately 193.2 g of EuCl_3_ (99% purity) can be recovered through this process. Table 3 summarizes the predicted revenue and expenses sheet for this system.

**Table 3:** Predicted cost/revenue to treat 1000 m^3^ of AMD

Item	Revenue (A$)	Expenses (A$)
Adsorbent preparation (1.3 kg)		2683.87
pH adjustment (NaOH)-industrial supplier		325.5
Desorption (HCl)-industrial supplier		312.55
Sale of EuCl_3_ (193.2 g)	11,881.90	
Sale of permeate water (800 m^3^)	2000.00	
**Total**	**13,881.90**	**3321.92**

Based on the above calculation, the membrane-adsorption hybrid system demonstrates a positive monetary return, excluding labor costs, membrane operation and maintenance costs. This discovery is particularly noteworthy, as there have been no previous studies evaluating the financial benefits of selectively recovering valuable metals. Further cost reductions are possible when purchasing chemicals in bulk. This system can also be expanded to recover other valuable metals such as Cu, potentially contributing to a significant increase in revenue as shown by Fonseka et al. (2022).

The cost–benefit analysis provided is preliminary and high-level, focusing on basic functions. A more detailed and comprehensive economic analysis is needed to fully understand the feasibility of mass production. Furthermore, this study did not account for potential market fluctuations in the prices of raw materials, processing costs, or the market value of recovered europium, which can impact economic feasibility.

## Conclusion

The practical application of recovering europium (Eu) from mining wastewater is a promising area of research. Given the increasing importance of rare earth elements (REEs) and the growing concerns about environmental sustainability, there is a considerable interest in developing efficient and cost-effective methods for recovering valuable metals from mining wastewater. This study evaluated the performance of two novel adsorbents, Cr-MIL-PMIDA and SBA15-NH-PMIDA, for recovering Eu from real mine wastewater. Initial equilibrium studies revealed that SBA15-NH-PMIDA has a 24% higher adsorption capacity than Cr-MIL-PMIDA. Furthermore, selective adsorption studies with real mining wastewater demonstrated similar selectivity for the two materials. However, the better reusability and lower production cost of SBA15-NH-PMIDA give it a broad applicability that transcends its slightly lower selectivity compared to Cr-MIL-PMIDA.

A cost–benefit analysis was conducted on these materials for the first time to quantify the advantages of using this novel material. The study revealed that 193.2 g of EuCl_3_ with 99% purity can be recovered by treating 1000 m^3^ of AMD. Clean water recovered from the membrane system can be reused in mining activities, mitigating water stress in arid regions. This highlights that by embracing resource recovery practices and technologies, the mining industry can move towards a more sustainable and circular approach to manage resources. This not only attracts financial benefits but also addresses environmental challenges, supports the transition to renewable energy, and fosters a more sustainable and equitable society. It is an essential component of achieving global sustainability goals and building a more resilient and regenerative future.

## Supplementary Information

Supplementary Material 1.

## Data Availability

The authors declare that the data supporting the findings of this study are available within the paper and its supplementary information files.
