# Supplementary material for: Selective recovery of europium from real acid mine drainage using modified Cr-MIL and SBA15 adsorbents
Source: Environ Sci Pollut Res Int. 2024 Aug 8;31(39):51540–50. doi: 10.1007/s11356-024-34566-2 (PMC11374818; doi:10.1007/s11356-024-34566-2)
Supplement: Supplementary file 1 — Supplementary Material 1. [file 11356_2024_34566_MOESM1_ESM.docx]

***Supporting Information***

**Selective recovery of Europium from real acid mine drainage using modified Cr-MIL and SBA15 adsorbents**

Charith Fonseka^a^, Seongchul Ryu^a^, Youngwoo Choo^a^, Jaya Kandasamy^a^, Lena Foseid^b^, Harsha Ratnaweera^b^, Saravanamuthu Vigneswaran^a,b *^

^a^ Department of Civil and Environmental Engineering, Faculty of Engineering and IT, University of Technology Sydney, P.O. Box 123, Broadway, Ultimo, NSW 2007, Sydney, Australia.

^b^ Department of Building and Environmental Technology, Faculty of Sciences & Technology (RealTek), Norwegian University of Life Sciences, P.O. Box N-1432, Oslo, Norway

*Corresponding author; Tel: +61-459-834-242, Email: [saravanamuth.vigneswaran@uts.edu.au](mailto:saravanamuth.vigneswaran@uts.edu.au)


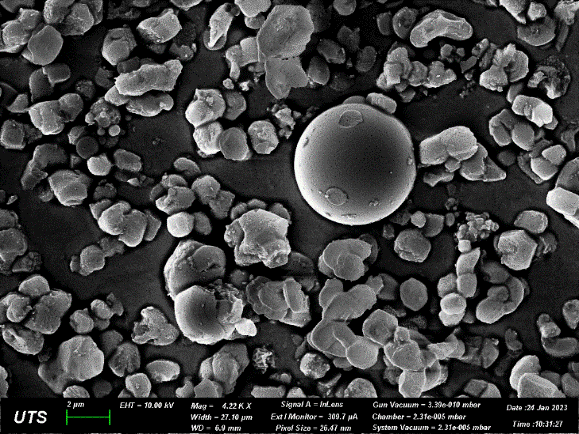

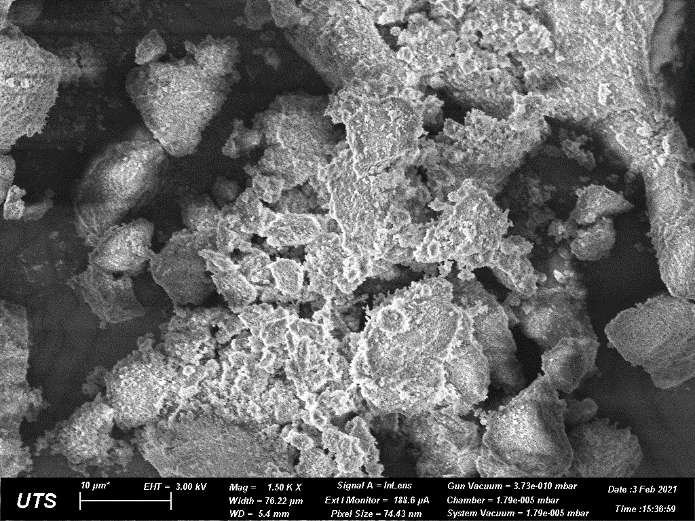


**(a)**

(b)

Figure S1: SEM image of a) SBA15-PMIDA and b) Cr-MIL-PMIDA


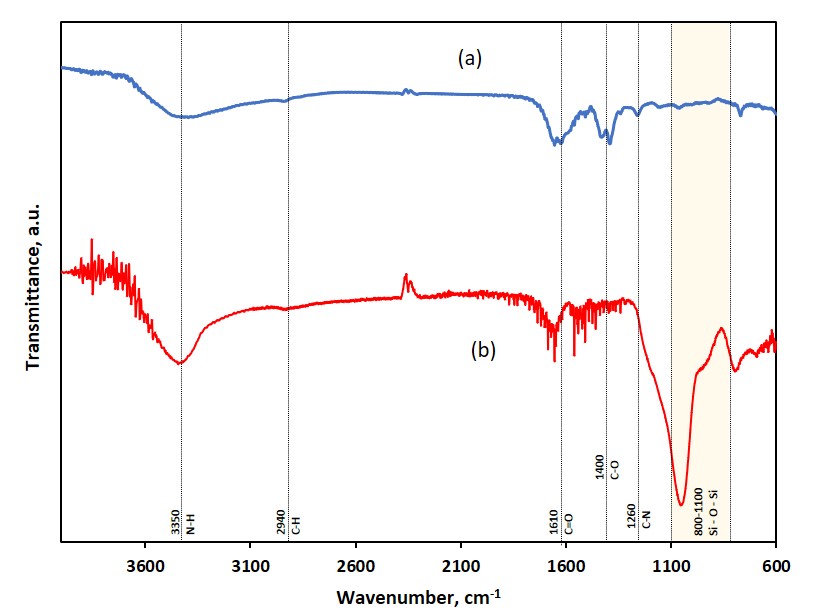


Figure S2: FT-IR spectra of (a) Cr-MIL-PMIDA and (b) SBA15-PMIDA


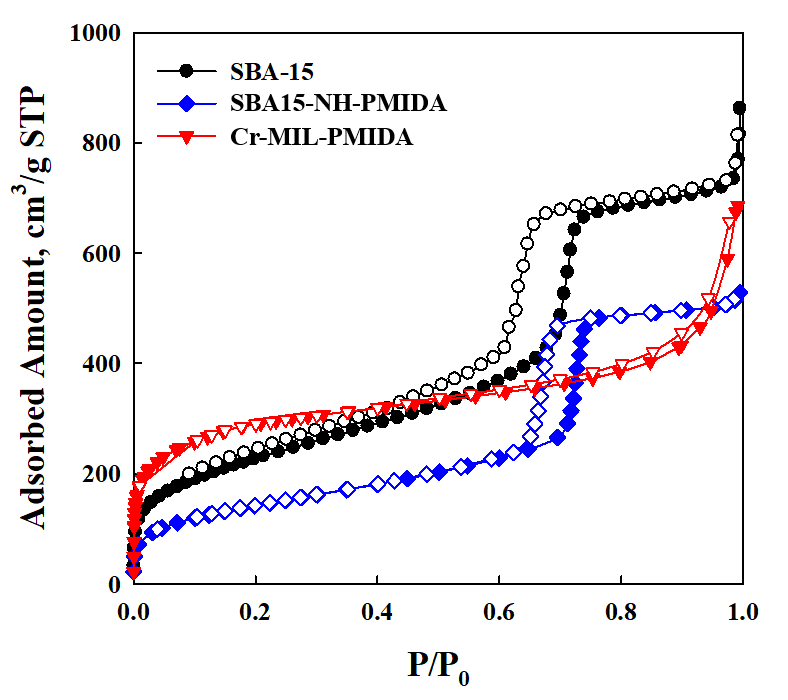


Figure S3: Nitrogen adsorption/desorption isotherm graphs of SBA-15, SBA15-NH-PMIDA and MIL-101-PMIDA

Table S1: Physical properties of adsorbents

| **Adsorbent** | **BET Surface Area** | **Pore Volume** | **Mean Pore Diameter** |
| --- | --- | --- | --- |
|  | (m^2^.g^-1^) | (cm^3^.g^-1^) | (nm) |
| Pristine SBA15 | 826 | 1.27 | 8.16 |
| SBA15-NH- PMIDA | 113 | 0.36 | 7.16 |
| Cr-MIL-PMIDA | 1049.4 | 0.69 | 2.15 |


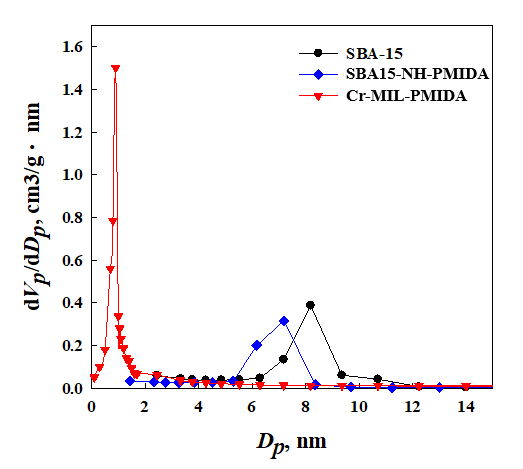


Figure S4: BJH pore size distributions of SBA-15, SBA15-NH-PMIDA and MIL-101-PMIDA

Table S2: Concentration of dissolved metals in AMD at different pH values (mg/l)

|  | | **Initial**  **(pH 2.0)** | **pH 3.0** | **pH 4.0** | **pH 4.5** | **pH 4.8** |
| --- | --- | --- | --- | --- | --- | --- |
| Mg | 220.0 ± 5.0 | | 190.0 ±7.0 | 150.0 ±5.0 | 130.0 ±5.0 | 130.0 ±5.0 |
| Al | | 170.0 ± 7.0 | 160.0 ±5.0 | 120.0 ±5.0 | 43.0 ±2.0 | ND |
| Ca | | 190.0 ± 5.0 | 180.0 ±5.0 | 165.0 ±5.0 | 110.0 ±5.0 | 110.0 ±5.0 |
| Cr | | 0.4 ± 0.1 | 0.4 ± 0.1 | 0.3 ± 0.1 | 0.1 ± 0.05 | ND |
| Mn | | 6.3 ± 0.3 | 5.8 ± 0.5 | 5.5 ± 0.3 | 3.5 ± 0.1 | 3.2 ± 0.1 |
| Fe | | 620.0 ± 5.0 | 580.0 ±7.0 | 58.0 ±5.0 | 9.7 ±1.0 | ND |
| Ni | | 0.5 ± 0.1 | 0.5 ± 0.1 | 0.5 ±0.1 | 0.3 ±0.1 | 0.3 ±0.1 |
| Cu | | 65.0 ± 2.0 | 53.0 ±3.0 | 40.0 ±3.0 | 31.0 ±3.0 | 24.0 ±2.0 |
| Zn | | 41.0 ± 3.0 | 36.0 ±3.0 | 25.0 ±2.0 | 23.0 ±3.0 | 24.0 ±2.0 |
| Eu | | 3.2 ± 0.1 | 2.8 ± 0.1 | 2.1 ± 0.1 | 1.4 ± 0.1 | 1.2 ± 0.1 |

**
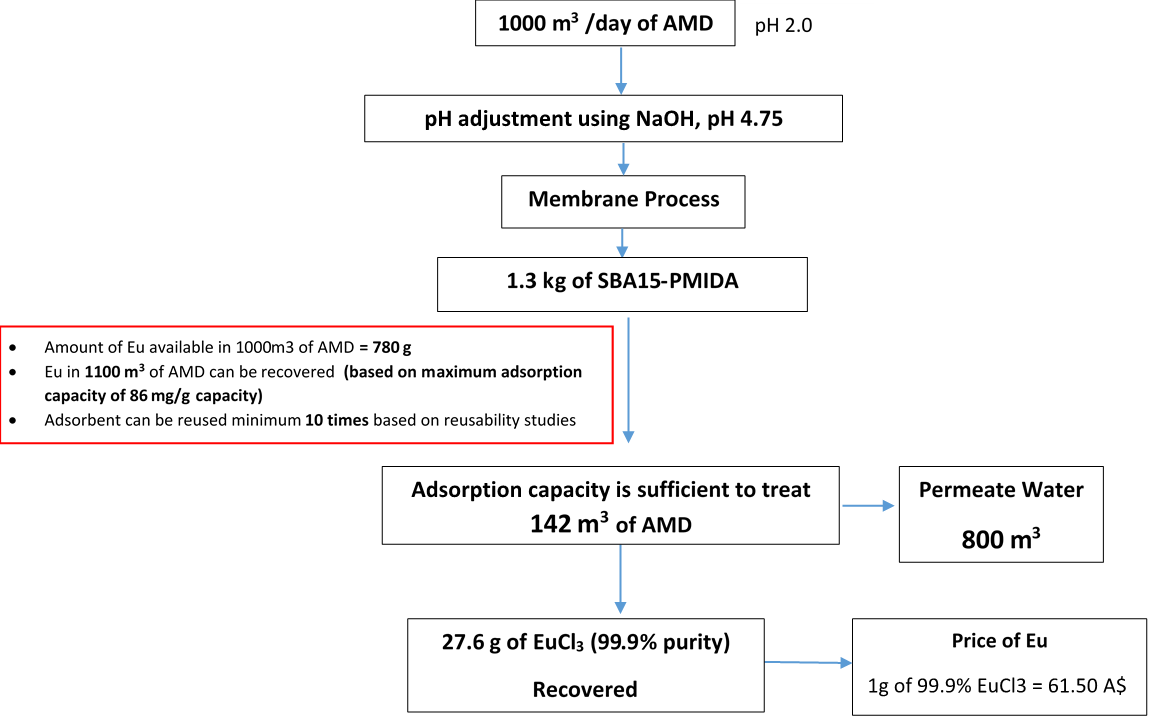
**

Figure S5: Estimated recovery of clean water and Eu from 1000 m^3^/day of AMD
